# Quantification of Rifaximin in Tablets by Spectrophotometric Method Ecofriendly in Ultraviolet Region

**DOI:** 10.1155/2016/3463405

**Published:** 2016-06-27

**Authors:** Ana Carolina Kogawa, Hérida Regina Nunes Salgado

**Affiliations:** Department of Pharmaceutics, School of Pharmaceutical Sciences of Araraquara, Univ Estadual Paulista (UNESP), Rodovia Araraquara-Jaú, km 1, 14801-902 Araraquara, SP, Brazil

## Abstract

Rifaximin is an oral nonabsorbable antibiotic that acts locally in the gastrointestinal tract with minimal systemic adverse effects. It does not have spectrophotometric method ecofriendly in the ultraviolet region described in official compendiums and literature. The analytical techniques for determination of rifaximin reported in the literature require large amount of time to release results and are significantly onerous. Furthermore, they use toxic reagents both for the operator and environment and, therefore, cannot be considered environmentally friendly analytical techniques. The objective of this study was to develop and validate an ecofriendly spectrophotometric method in the ultraviolet region to quantify rifaximin in tablets. The method was validated, showing linearity, selectivity, precision, accuracy, and robustness. It was linear over the concentration range of 10–30 mg L^−1^ with correlation coefficients greater than 0.9999 and limits of detection and quantification of 1.39 and 4.22 mg L^−1^, respectively. The validated method is useful and applied for the routine quality control of rifaximin, since it is simple with inexpensive conditions and fast in the release of results, optimizes analysts and equipment, and uses environmentally friendly solvents, being considered a green method, which does not prejudice either the operator or the environment.

## 1. Introduction

**Figure 1:**
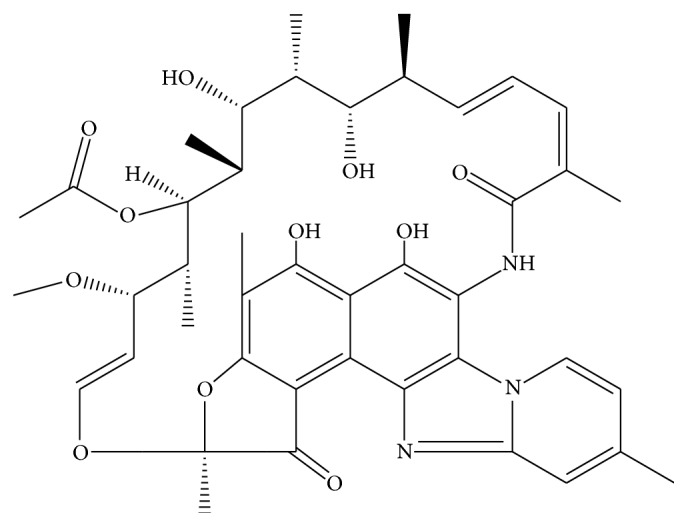
Chemical structure of rifaximin (CAS 80621-81-4).

Rifaximin (Figure 1) is an oral antibiotic with broad spectrum of action and nonabsorbable that acts locally in the gastrointestinal tract with minimal systemic adverse effects [1, 2]. It is practically not absorbed and reaches high concentrations in the human intestine, where it is active against many enteropathogens [3].

Rifaximin (C_43_H_51_N_3_O_11_ and molecular weight 785.9 g mol^−1^) is a derivative of rifamycin and is a structural analogue of rifampicin.

The quality of a pharmaceutical product is directly related to the health of patients and a practical and accurate analysis method can be the first step in the rational use of pharmaceuticals [4].

During the development of a new analytical method, various factors must be considered such as reliability, detection, and separation of all compounds of interest, analysis speed to optimize equipment and analysts, reduced need for pretreatment of the sample, low final cost of analysis by accounting reagents, procedures and equipment, and use of nontoxic reagents for neither the operator nor the environment [5, 6].

The introduction of analytical methods requires the use of validation procedures that confer reliability required for the application of quantification techniques. Validation is the process which defines an analytical exigency and confirms that the method under investigation has performance capabilities consistent with what the application requires [7].

Universities have executed a crucial role serving as research centers for the development and validation of analytical methodologies, contributing to health control activities and scientific enrichment in the area [8].

Rifaximin does not have spectrophotometric method in the ultraviolet region described in official compendiums. In the literature, an article which describes a spectrophotometric method in the ultraviolet region has been found; however this method uses buffer and methanol [9]. The advantage of our proposal is exactly to avoid the use of toxic reagents for both the operator and environment. Furthermore, our proposal also minimizes the use of reagents and stages, reaching analysis with rapid release of results.

Spectrophotometric method in the ultraviolet region is a technique easy to perform and with reduced cost. It can be used with good precision to the quantitative analysis of drugs present in pharmaceutical formulations, since there is no interference of excipients [10, 11].

The analytical techniques for determination of rifaximin reported in the literature utilize liquid chromatography that require large amount of time to release results and are significantly onerous [12–18]. Furthermore, they use toxic reagents for both the operator and environment and, therefore, cannot be considered environmentally friendly analytical techniques.

The objective of this study was to develop and validate an ecofriendly spectrophotometric method in the ultraviolet region to quantify rifaximin in tablets.

## 2. Experiments

### 2.1. Chemicals and Reagents

The reference standard (secondary standard) was rifaximin, content 99.0%, acquired from the company NutraTech Development Limited (China).

The pharmaceutical form was rifaximin tablets of 200 mg (labeled content), lot 12927, under the trade name Flonorm*™*, of the Laboratory Gonher Farmaceutica LTDA.

In the development of method, the placebo was prepared using quantities of excipients usually present in a tablet. However, the concentration of red iron oxide was increased, in order to observe the interference in analytical response of method.

The placebo preparation was carried out by physical mixing of excipients, described in the following proportions:

 Sodium carboxymethyl (0.18%).
 Glyceryl palmitostearate (1.5%).
 Colloidal anhydrous silica (1.0%).
 Talc (30%).
 Microcrystalline cellulose (20%).
 Hypromellose (0.4%).
 Titanium dioxide (1.4%).
 Disodium edetate (0.18%).
 Propylene glycol (0.3%).
 Red iron oxide (1.0%).

The reagents used were purified water (Millipore*™*) and ethyl alcohol (Qhemis*™*).

### 2.2. Equipment

The equipment used was Spectrophotometer UV mini-1240 (Shimadzu*™*), quartz cuvettes with 1 cm optical path, analytical balance model DV215CD (Ohaus*™*), water purification system (Millipore), and ultrasound equipment Ultrasonic Cleaner (Unique*™*).

### 2.3. Spectrophotometric Method in the UV Region

#### 2.3.1. Development

20.00 mg of standard was weighed and transferred to a 100 mL volumetric flask completing the volume with initial 20 mL of ethanol and then purified water to give concentration of 200 *μ*g mL^−1^ in purified water with 20% ethyl alcohol. About 50 mL of this stock solution was filtered through filter paper and, from this solution, 1.1 mL aliquot was transferred to a 10 mL volumetric flask, completing the volume with purified water and 20% ethyl alcohol to yield a final concentration of 22 *μ*g mL^−1^.

The average weight from twenty tablets of rifaximin was 373.89 mg. Subsequently, the tablets were triturated and weighed 18.69 mg of rifaximin sample (equivalent to 10 mg standard). This amount was transferred to 100 mL volumetric flask completing the volume with initial 20 mL of ethanol and then purified water to give a concentration of 100 *μ*g mL^−1^ in purified water with 20% ethyl alcohol. About 50 mL of this stock solution was filtered through filter paper and, from this solution, 2.2 mL was transferred to a 10 mL volumetric flask, completing the volume with purified water and 20% ethyl alcohol to yield a final concentration of 22 *μ*g mL^−1^.

The placebo was analyzed with the standard and sample to evaluate its influence on the spectrophotometric analysis in the UV region. 8.69 mg of placebo (equivalent to the amount present in 10 mg sample) was weighed and transferred to a 100 mL volumetric flask completing the volume with initial 20 mL of ethanol and then purified water, obtaining the same concentration of placebo that would be present in the sample solution. About 50 mL of this stock solution was filtered through filter paper and, from this solution, 2.2 mL was transferred to a 10 mL volumetric flask, completing the volume with purified water and 20% ethyl alcohol to yield a final concentration of 22 *μ*g mL^−1^.

Spectra were obtained from the scanning in the wavelength range 200 to 400 nm.

The identification of standard and tablets, as well as evaluating the influence of placebo, was performed by overlapping the spectra obtained and comparison as to their characteristic profile and absorption wavelengths.

#### 2.3.2. Validation

*(1) Ringbom Curve.* The Ringbom curve was obtained by the determination of absorbance of 43 standard concentrations of rifaximin at wavelength 290 nm.

20 mg of standard rifaximin was weighed and transferred to a 100 mL volumetric flask. The initial substance was dissolved in 20 mL of ethanol and then purified water and kept in ultrasound for 15 minutes. The flask volume was completed to obtain a solution with final concentration 200 *μ*g mL^−1^. From this solution, aliquots were transferred to volumetric flasks, yielding a curve with 43 points, with concentrations varying from 1 to 200 *μ*g mL^−1^ of rifaximin.

*(2) Validation Parameters. *Validation parameters were as follows.

*Linearity. *From the Ringbom curve, 6 points were chosen for evaluation of the linearity of the method. For this, 10 mg of rifaximin working standard was weighed, transferred to a 100 mL volumetric flask, dissolved in initial 20 mL of ethanol and then purified water, and kept in ultrasonic equipment for 15 minutes. Solutions with concentrations of 10, 14, 18, 22, 26, and 30 *μ*g mL^−1^ of standard rifaximin were obtained with the use of automatic pipettes. The readings of the solutions were made at 290 nm, using purified water with 20% ethanol as blank. The analytical curve was constructed on three different days and in triplicate.

The data obtained in the construction of the calibration curve were analyzed to obtain the equation of the least squares method and the check of linearity and parallelism was confirmed by Analysis of Variance (ANOVA).

The linearity is the ability of an analytical method to demonstrate that the results obtained are directly proportional to the concentration of analyte in the sample, within a specified range. It is recommended that linearity be determined by analysis of, at least, 5 different concentrations. If there is apparent linear relationship after visual examination of the graph, the test results should be processed by appropriate statistical methods to determine the correlation coefficient (*r*), intersection with the *y*-axis, angular coefficient, residual sum of least squares of the linear regression, and relative standard deviation (RSD). The minimum criteria acceptable for *r* must be equal to 0.99.

*Limit of Detection and Limit of Quantification*. Limit of detection (LD) of rifaximin was determined from the three calibration curves obtained, using the dates of standard deviation of the intercept (*s*) and average slope (*l*), according to

(1) $$\begin{array}{c} LD=\frac{\left(3.3s\right)}{l}. \end{array}$$

LD is the lowest content that can be measured with reasonable statistical certainty [18]. It is the lowest concentration of analyte in a sample that can be detected but not necessarily quantitated under the test established conditions [19].

Limit of quantification (LQ) of rifaximin was obtained in the same data described above, according to

(2) $$\begin{array}{c} LQ=\frac{\left(10s\right)}{l}. \end{array}$$

LQ is the content equal to or higher than the lower concentration point in analytical curve [20] or the lowest concentration of analyte in a sample which can be determined with acceptable precision (repeatability) and accuracy under the conditions of the test [19].

*Selectivity.* The selectivity of the proposed method was confirmed by comparison of the response obtained for the active substance in the absence of adjuvants (standard) and that in the presence of adjuvants (tablets).

Selectivity was also proven by the forced degradation of rifaximin in acidic conditions using HCl 0.01 M, basic conditions using NaOH 0.001 M, oxidative conditions using H_2_O_2_ 10%, neutral conditions using H_2_O, and photolytic using UV light.

The selectivity is the capacity of the method measure of exactly a compound in the presence of other components such as impurities, degradation products, and matrix components.

*Precision*. Precision was tested by realization of the absorbance reading of rifaximin at a concentration of 22 *μ*g mL^−1^ six times on the same day and on different days, to evaluate the intraday and interday precision, respectively.

The results were evaluated by the percentage of the RSD, according to

(3) $$\begin{array}{c} RSD\left(\;\%\;\right)=\frac{SD}{ACD}\times 100, \end{array}$$

where SD is standard deviation and ACD is average concentration determined.

Precision is the evaluation of proximity of the results obtained in a series of multiple measurements of the same sample. It can be classified as repeatability or intraday precision and intermediate or interday precision. Repeatability is the concordance between the results within a short period of time with the same analyst and the same instrumentation. Intermediate precision is the concordance between the results from the same laboratory, but obtained on different days, with different analysts. The precision of an analytical method can be expressed as the RSD of a series of measures. The acceptable maximum value must be defined according to the methodology employed, the concentration of the analyte in the sample, and the purpose of the method, not accepting values above 5%.

*Accuracy.* The accuracy of the method was demonstrated by recovery test.

Stock solutions of rifaximin standard and sample were prepared at concentration of 100 *μ*g mL^−1^. Of these solutions, 500 *μ*L aliquots of each were taken and transferred to 5 mL volumetric flask, obtaining two volumetric flasks with 500 *μ*L each, one of standard and the other of sample. They were completed with purified water and 20% ethyl alcohol and obtained theoretical solutions with concentrations of 10 *μ*g mL^−1^. From the rifaximin sample stock solution, 500 *μ*L aliquots were taken and transferred to 5 mL volumetric flask added to 300 *μ*L of rifaximin standard stock solution, completed with purified water and 20% ethyl alcohol to obtain solution with theoretical concentration of 16 *μ*g mL^−1^. Changing only the volume of standard stock solution was carried out similarly to obtain two solutions through aliquots of 500 *μ*L (theoretical concentration of 20 *μ*g mL^−1^) and 700 *μ*L (theoretical concentration of 24 *μ*g mL^−1^).

The preparations of the samples are exemplified in Table 1.

The percentage of recovered rifaximin was calculated by [18]

(4) $$\begin{array}{c} \%\;\;R=\left\{\;\frac{\left(C_{r}-C_{a}\right)}{C_{p}}\;\right\}\times 100, \end{array}$$

where *C*_*r*_ is concentration of sample solution added of standard (*μ*g mL^−1^), *C*_*a*_ is concentration of sample solution (*μ*g mL^−1^), and *C*_*p*_ is theoretical concentration of standard solution added (*μ*g mL^−1^).

The accuracy of an analytical method is the closeness of the results obtained by the method under study in relation to the true value. It can be determined by standard addition method, where known amounts of the standard are added, and by contaminated placebo method, in which drug known amount is added to a mixture of the medicament components. The accuracy is calculated as percentage of recovery of the known amount of the reference standard added to the sample.

*Robustness.* The robustness of the method was determined by comparison of the results obtained by variation of two units of wavelength above and below 290 nm, 288 nm, and 292 nm, by source of purified water, Laboratory A and Laboratory B, by percentage of ethyl alcohol, 20% and 18%, and by ultrasound time at which the solutions were submitted, 15 and 10 minutes. The results obtained by variation of this operating parameters were analyzed by *F* test (Snedecor) and *t*-test (Student).

The robustness of an analytical method is the measure of its ability to resist small changes of the analytical parameters. It indicates the confidence during the routine use. In evaluating the robustness, the susceptibility of the method to variations in analytical conditions is observed and, thus, the precautions must be included in the procedure to control the process.

#### 2.3.3. Content Analysis

The rifaximin content in tablets was determined by weighing sample equivalent to 10 mg of rifaximin standard. In a pool of 20 tablets of rifaximin crushed, 18.69 mg of sample was weighed and transferred to a 100 mL volumetric flask and then dissolved in initial 20 mL of ethyl alcohol and then purified water kept in ultrasound for 15 minutes. The volume of volumetric flask was completed, obtaining solution with final concentration of 100 *μ*g mL^−1^. Approximately 50 mL of this stock solution was filtered through filter paper and from this solution 1.1 mL aliquot was transferred to 5 mL volumetric flask to give a final concentration of 22 *μ*g mL^−1^.

The calculation of concentration and rifaximin content in the solutions was performed by (5) and (6), respectively. Consider

(5) $$\begin{array}{c} C_{a}=A_{a}\times \frac{C_{p}}{A_{p}}, \end{array}$$

where *C*_*a*_ is concentration of the sample solution (*μ*g mL^−1^), *A*_*a*_ is absorbance of the sample solution, *C*_*p*_ is theoretical concentration of the standard solution (*μ*g mL^−1^), and *A*_*p*_ is absorbance of the standard solution. Consider

(6) $$\begin{array}{c} C_{a}\%=\frac{\left(C_{a}\times 100\right)}{C_{t}}, \end{array}$$

where *C*_*a*_% is percentage concentration of rifaximin in the solutions, *C*_*a*_ is rifaximin concentration found in the solutions (*μ*g mL^−1^), and *C*_*t*_ is theoretical concentration of rifaximin in the solutions (*μ*g mL^−1^).

## 3. Results

### 3.1. Development

**Figure 2:**
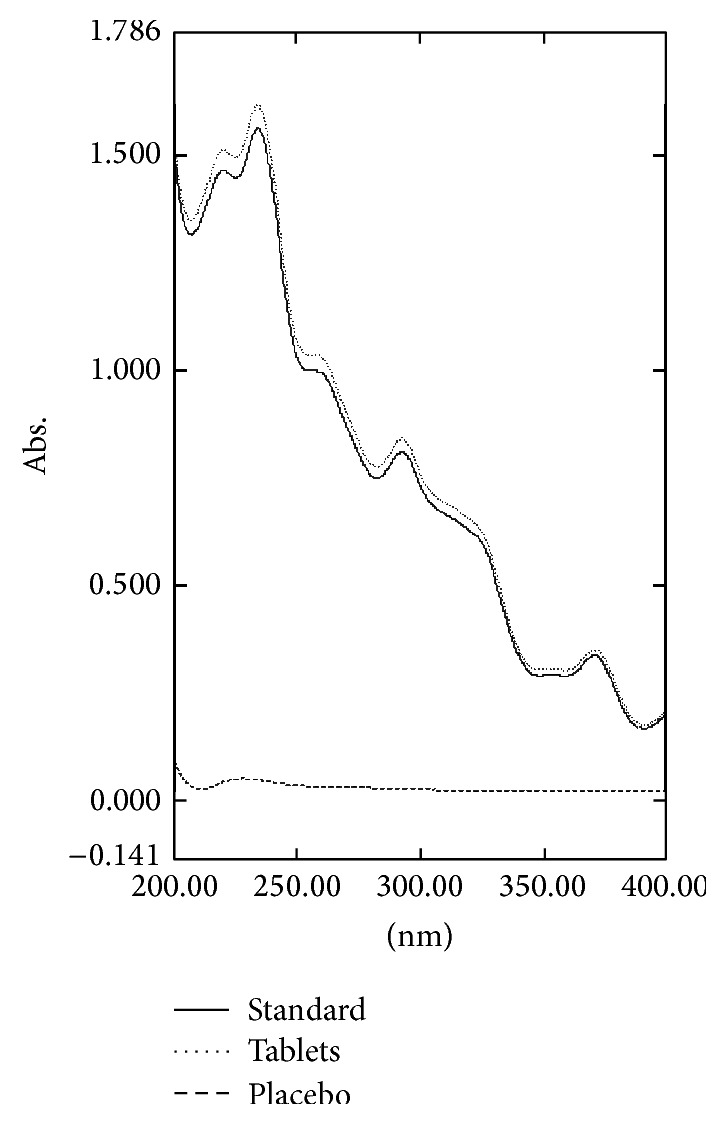
Overlap of standard, tablets, and placebo spectra, in purified water with 20% ethanol at concentration of 22 *μ*g mL^−1^.

Figure 2 shows the overlap of standard, tablets, and placebo spectra, in purified water with 20% ethanol at concentration of 22 *μ*g mL^−1^. It is possible to observe that the adjuvants of the tablets do not have absorption in the wavelength used in the spectrophotometric analysis in the UV region.

### 3.2. Validation

#### 3.2.1. Ringbom Curve

**Figure 3:**
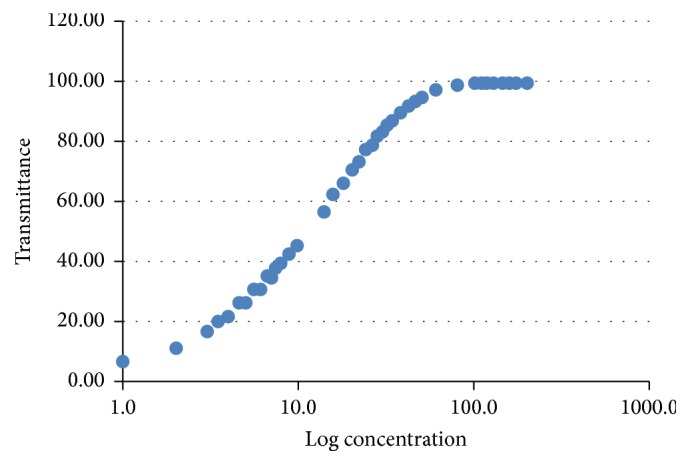
Ringbom curve for rifaximin working standard.

The absorbance values obtained for different concentrations of standard rifaximin to obtain the Ringbom curve at wavelength 290 nm are shown in Figure 3.

#### 3.2.2. Validation Parameters

**Figure 4:**
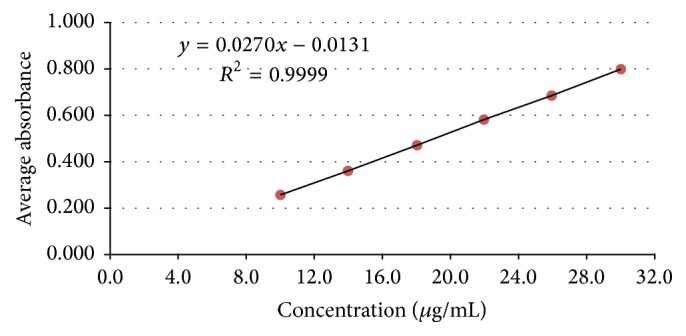
Analytical curve of standard rifaximin, at concentrations of 10, 14, 18, 22, 26, and 30 *μ*g mL^−1^, obtained by the spectrophotometry method in the ultraviolet region in purified water with 20% ethyl alcohol.

*Linearity.* The analytical curve of standard rifaximin was constructed with the average of the absorbance values of three calibration curves at concentrations from 10 to 30 *μ*g mL^−1^.  Figure 4 shows the corresponding calibration curve.

The equation of the line, determined by the method of least squares, is *y* = 0.0270*x* + 0.0131, with coefficient of determination (*R* ^2^) equal to 0.9999 and correlation coefficient (*r*) greater than 0.9999 for rifaximin standard work.

The ANOVA calculated for the data of the analytical curves of standard rifaximin is shown in Table 2.

*Limit of Detection and Limit of Quantification.* The results of limit of detection and limit of quantitation were 1.39 *μ*g mL^−1^ and 4.22 *μ*g mL^−1^, respectively.

**Figure 5:**
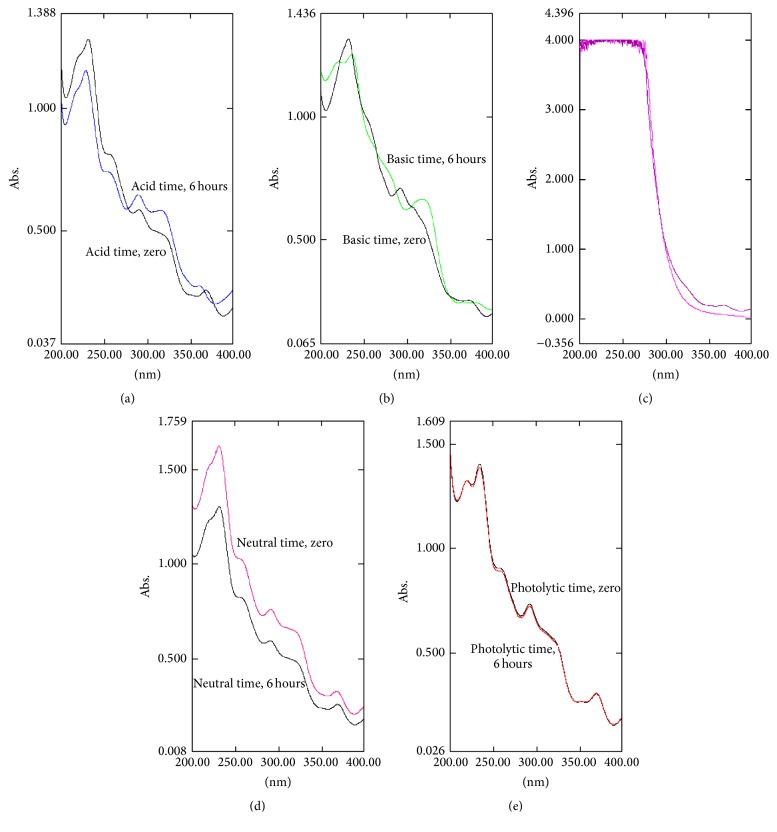
Absorption spectra obtained by absorption spectrophotometric in the ultraviolet region for sample solutions of rifaximin subjected to the following conditions: (a) acid, (b) basic, (c) oxidative, (d) neutral, and (e) photolytic at a concentration of 22 *μ*g mL^−1^.

*Selectivity.* Figure 5 shows the degradation of rifaximin under stress conditions.

*Precision.* Intraday and interday precision were made by repeatability and intermediate precision, respectively. The results are shown in Table 3.

*Accuracy.* Table 4 shows the recovery values obtained for each concentration level tested by absorption spectrophotometry method in the ultraviolet region.

*Robustness.* The averages of changes, at the wavelength, in the source of purified water, and in the percentage of ethyl alcohol used in the diluent and ultrasound time by which the solutions were subjected, to evaluate the robustness parameter were statistically analyzed and the results are shown in Table 5.

### 3.3. Content Analysis

The assay was performed by comparing the absorbance values obtained in the analysis of the standard solutions of rifaximin and sample solutions of rifaximin, both at a concentration of 22 *μ*g mL^−1^. The results are shown in Table 6.

## 4. Discussion

The spectrophotometry in the UV region has a variety of applications in pharmaceutical analysis. The identification of a drug can be made through the analysis of its absorption spectrum in the UV region (200 to 400 nm). It is a useful method for identification and quantification of drugs. It is a rapid assay with release of results in an instant, simple by requiring only the cuvette as apparatus, and easy to be performed.

The quick release of results optimizes analysts, who can attend to other tasks, and equipment, which will be available for another analysis. This fact in laboratory or industrial scale makes a big difference at the end of the day, month, or year. The same thought occurs in the choice of reagents for an analysis. If they are cheap the cost of analysis becomes smaller and, moreover, if they are green reagent there is no need for additional cost to make the disposal of chlorinated, organic, or explosive substances.

Rifaximin has various nitrogen atoms in its structure and its theoretical pKa is 5.14. An increase in pH of the solution for the solubilization of rifaximin, thus, promotes an increase in the number of molecules of rifaximin protonated at functional groups containing nitrogen atoms, making, thereby, rifaximin more soluble at higher pHs. Conversely, a decrease in pH tends to decrease the solubility of rifaximin. Ethanol solubilized rifaximin to be less polar than water, interacting, thus, more effectively with the structure relatively lipophilic of rifaximin. The solvent of choice for spectrophotometric determination was, then, purified water with 20% ethyl alcohol, because the aim of this study was to develop a simple and low cost method, using green solvents with low toxicity for the operator and for the environment.

Ethanol is considered environmentally friendly, a green solvent, and ecofriendly which does not affect the operator or the environment and can be handled without risk and disposed untreated [21–23].

The purified water cannot be used only for the solubilization of rifaximin for not being soluble in this solvent. Buffers solutions in pH that varied from pH of 3.0 to 9.0 showed no complete solubilization of rifaximin, even when a solubilizing agent was added to the solution.

The absorption spectra obtained in the standard and sample analysis of rifaximin showed similar profiles, suggesting the same identity of the samples. Both the standard and the sample exhibited absorption at 232 and 290 nm when using purified water with 20% ethanol as solvent. The placebo of rifaximin tablets had no effect on spectrophotometric analysis of the drug in the UV region. Thus, the method proved to be helpful in the identification of rifaximin in tablets.

The validation of analytical methods is required as a fundamental requirement in the accreditation for quality assurance and demonstration of technical competence. The validation must guarantee, through experimental studies, that the method meets the requirements of the analytical applications, ensuring the reliability of the results. For this, it must present aspects of linearity, specificity, accuracy, precision, limits of detection, and quantification and robustness appropriate for analysis.

These analytical parameters were evaluated for the validation of the method according to RE number 899 [24], AOAC [20], ICH [25], and INMETRO [26].

In the development of the spectrophotometric method in the UV region to rifaximin concentrations ranging from 1 to 200 *μ*g mL^−1^ were tested. The concentrations from 10 to 30 *μ*g mL^−1^ were chosen, since they had better linearity of response.

To evaluate the linearity of the method the graph concentration versus absorbance was constructed, which demonstrated proportionality between these two factors. The equation of the line was *y* = 0.0270*x* + 0.0131 and correlation coefficient was 0.9999. The linearity was evidenced by ANOVA, which showed that there was regression between concentrations and linearity deviation not significant.

The low values calculated for the limits of detection and quantification of rifaximin (LD = 1.39 *μ*g mL^−1^ and LQ = 4.22 *μ*g mL^−1^) indicate the good sensitivity of the method for the determination of the drug.

The selectivity of the method was confirmed by comparison of the absorbance values of the standard and sample. The spectra of solutions of rifaximin standard and tablets were analyzed in relation to possible interference of excipients at the wavelength used. The result of the overlap of the spectra showed that the excipients do not interfere with the readings of the analyses.

The selectivity of the method also was confirmed by stress study, in which rifaximin exposed to acidic, basic, oxidative, neutral, and photolytic conditions was evaluated. The drug profile at time zero (no exposure to degrading conditions) is compared to the time 6 hours of exposure to these conditions. The profile of rifaximin changes according to drug degradation occurs, showing specific selectivity of the method.

The method proved to be precise presenting deviation values for intraday precision (0.57%) and interday precision (1.08%) lower than 2%.

Accuracy of the method is defined as the correlation between the result of a test and the reference value conventionally accepted as true. The accuracy when applied to a series of test results involves a combination of random and systematic errors [26].

Thus, the accuracy is always associated with precision values; these limits can be narrow in levels of concentration high and broader in levels of traits, following the example of Horwitz studies [27, 28].

Horwitz and coworkers (1980) established a mathematical relationship to express the dependency between the RSD values and analyte concentration, by exam of cumulative results of collaborative studies involving a wide range of compounds. The values obtained by this mathematical relationship were laid out in a graph and gave rise to the so-called Horwitz Trumpet [27].

There are acceptable critical values according to the concentration of the analyte under study. These values are estimated considering the analysis that major elements often have systematic errors much lower than those obtained for analytes in very low concentrations. These values, suggested by the manual of the Association of Official Analytical Chemists (AOAC), are presented in Table 7.

The concentration of rifaximin in assay of accuracy was between 37.5 and 58.3% and, in this case, the acceptable recovery interval is 98 to 102%. Thus, the method can be considered accurate for presenting an average of 100.17%.

The method was robust in the face of small variations in wavelength used, purified water source used in the analysis, and the percentage of ethyl alcohol used to solubilize the samples. However, the method has not kept robustness when the ultrasound time decreased from 15 minutes to 10 minutes. The time of 15 minutes proved to be crucial for the complete solubilization of the drug, which is of fundamental importance to the rigor of this parameter in the reproduction of this method.

The amount of rifaximin present in the analyzed samples is 109.28%.

## 5. Conclusions

A spectrophotometric method in the UV region has been developed and validated for the quantification of rifaximin in tablets. It showed linearity, selectivity, accuracy, precision, robustness, and adequate detection and quantification limits in the range of 10 to 30 *μ*g mL^−1^. Its advantages are simplicity, inexpensive conditions, speed in the release of results, optimization analysts and equipment, and use of environmentally friendly solvents, being considered a green method, which does not prejudice either the operator or the environment.

## Figures and Tables

**Table 1:** Preparation of the solutions to the accuracy test of the spectrophotometric method in the UV region.

	Volume added of rifaximin sample of concentration 100 *μ*g mL^−1^ (*µ*L)	Volume added of rifaximin standard of concentration 100 *μ*g mL^−1^ (*µ*L)	Final theoretical concentration (*μ*g mL^−1^)^a,b^
Sample	500	—	10
R1	500	300	16
R2	500	500	20
R3	500	700	24
Standard	—	500	10

^a^Diluted in 5 mL volumetric flask.

^b^Each concentration level was prepared in triplicate.

**Table 2:** Analysis of variance of the absorbance values determined in obtaining of the analytical curves of standard rifaximin, using absorption spectrophotometry method in the ultraviolet region.

Variation sources	DF	SS	Variance	*F*_cal_	*F*_tab_ (0.05)
Among concentrations	5	0.61	0.12	140.45^*∗*^	3.11
Linear regression	1	0.61	0.61	702.16^*∗*^	4.75
Linearity deviation	4	0.00	0.00	0.02	3.26
Inside (waste)	12	0.00	0.00	—	—

Total	17	0.61	—	—	—

^*∗*^Significant for *p* < 5%.

DF: degrees of freedom; SS: sum of squares.

**Table 3:** Values determined for the parameter precision of rifaximin work standard by absorption spectrophotometric method in the ultraviolet region.

Concentration (*µ*g mL^−1^)	Test	Absorbance values						RSD (%)
		1	2	3	4	5	6	
22	Repeatability	0.581	0.587	0.583	0.578	0.586	0.582	0.57 (*n* = 6)
	Intermediate precision	0.584	0.568	0.575	0.574	0.566	0.563	1.08 (*n* = 12)
		0.579	0.580	0.582	0.577	0.561	0.582	

**Table 4:** Values of recovery test of the absorption spectrophotometric method in the ultraviolet region.

	Standard rifaximin added (*µ*g mL^−1^)	Standard rifaximin recovered (*µ*g mL^−1^)	Recovery (%)	Average recovery (%)	RSD (%)
R1	6	6.01	100.18	100.17	
R2	10	10.03	100.32		0.15
R3	14	14.00	100.02		

**Table 5:** Calculated values through the *F* and *t*-tests to variations of absorption spectrophotometric method in the ultraviolet region for determination of rifaximin in tablet.

Test	Wavelength (nm)		Wavelength (nm)		Source of purified water		Ethyl alcohol (%)		Ultrasound time (min)	
	290	288	290	292	Lab A	Lab B	20	18	15	10
*F*_cal_	1.0		1.11		1.48		9.81		10.36	
*F*_tab_	19.0		19.0		19.0		19.0		19.0	
*t*_cal_	0.47		0.75		0.24		2.08		5.41	
*t*_tab_	2.78		2.78		2.78		2.78		2.78	

**Table 6:** Values determined for the assay of rifaximin in tablets by absorption spectrophotometric method in the ultraviolet region.

Day	Rifaximin content^a^		Average	RSD (%)
	*µ*g mL^−1^	%		
1	23.53	106.95	109.28	3.52
2	23.58	107.16		
3	25.02	113.71		

^a^Each value represents the average of three determinations.

**Table 7:** Recovery of analyte according to the concentration.

Analyte concentration (%)	Recovery interval accepted (%)
≥10	98–102
≥1	97–103
≥0.1	95–105
≥0.01	90–107
≥0.001–≥0.00001	80–110
≥0.000001	60–115
≥0.0000001	40–120
